# Supplementary material for: COVID-19 vaccine hesitancy and confidence in the Philippines and Malaysia: A cross-sectional study of sociodemographic factors and digital health literacy
Source: PLOS Glob Public Health. 2022 Oct 19;2(10):e0000742. doi: 10.1371/journal.pgph.0000742 (PMC10021455; doi:10.1371/journal.pgph.0000742)
Supplement: Attachment — Submitted filename: Response to Reviewers.docx [file pgph.0000742.s002.docx]

September 1 2022

*PLoS One Global Public Health*

Dear reviewers,

Please accept our sincerest gratitude for your time and effort in providing thoughtful feedback on our manuscript. Your responses has helped us in transforming this manuscript for the better – and we gratefully appreciate it.

Please find the following comments to your kind suggestions.

Warm wishes,

Dr. Ken Brackstone, Ph.D.

University of Southampton

Clinical Informatics Research Unit,

Care of the National Blood and Transplant Service, MP 852,

Coxford Road,

Southampton, UK

SO16 5AF

**Journal requirements:**

1. Please change "female” or "male" to "woman” or "man" as appropriate, when used as a noun (see for instance [https://apastyle.apa.org/style-grammar-guidelines/bias-free-language/gender](https://eur03.safelinks.protection.outlook.com/?url=https%3A%2F%2Fapastyle.apa.org%2Fstyle-grammar-guidelines%2Fbias-free-language%2Fgender&data=05%7C01%7CK.Brackstone%40soton.ac.uk%7C111a64f09b774a484bb108da89f683de%7C4a5378f929f44d3ebe89669d03ada9d8%7C0%7C0%7C637973989131455106%7CUnknown%7CTWFpbGZsb3d8eyJWIjoiMC4wLjAwMDAiLCJQIjoiV2luMzIiLCJBTiI6Ik1haWwiLCJXVCI6Mn0%3D%7C3000%7C%7C%7C&sdata=65IyXWgYP6%2FAJy%2F3ucngr0Xs8%2FmD8NPL1z2wWo4LE5g%3D&reserved=0)).

Sure – thank you. We have now amended the manuscript and tables to include woman/women and man/men, where appropriate.

2. Please include a complete copy of PLOS’ questionnaire on inclusivity in global research in your revised manuscript. Our policy for research in this area aims to improve transparency in the reporting of research performed outside of researchers’ own country or community. The policy applies to researchers who have travelled to a different country to conduct research, research with Indigenous populations or their lands, and research on cultural artefacts. The questionnaire can also be requested at the journal’s discretion for any other submissions, even if these conditions are not met.  Please find more information on the policy and a link to download a blank copy of the questionnaire here: [https://journals.plos.org/globalpublichealth/s/best-practices-in-research-reporting](https://eur03.safelinks.protection.outlook.com/?url=https%3A%2F%2Fjournals.plos.org%2Fglobalpublichealth%2Fs%2Fbest-practices-in-research-reporting&data=05%7C01%7CK.Brackstone%40soton.ac.uk%7C111a64f09b774a484bb108da89f683de%7C4a5378f929f44d3ebe89669d03ada9d8%7C0%7C0%7C637973989131455106%7CUnknown%7CTWFpbGZsb3d8eyJWIjoiMC4wLjAwMDAiLCJQIjoiV2luMzIiLCJBTiI6Ik1haWwiLCJXVCI6Mn0%3D%7C3000%7C%7C%7C&sdata=ib4VeOUnwWb2VTsBoDx4%2BYlbMZokcyl6N4MF1mfNTEY%3D&reserved=0). Please upload a completed version of your questionnaire as Supporting Information when you resubmit your manuscript.

Thanks. Please find a copy of this questionnaire enclosed to this submission.

**Reviewer #1:**

1.The author should indicate the dates the following references were retrieved: 12, 13, 14, 15, 16,17,18,9, 22,25,26,27,28,29-30,33, 36,37.

Many thanks for listing these references and making them easy for us to identify. Dates have now been added to the relevant references. Although, the majority of these references required digital object identifiers, so these have now been added. We have also tidied up the references section as a whole.

2. The author should add ''Implication of the study'' as well as ''Contribution to body of Knowledge''

Thank you. The second to last paragraph before the conclusion contains important implications for health authorities and governments in areas focusing on improving vaccination uptake. These have now been explicitly highlighted as implications at the beginning of this paragraph (in the Discussion).

**Reviewer #2:**

• Vaccine hesitancy and vaccine confidence are related but distinct constructs. The author(s) define vaccine hesitancy, but no definition is provided for vaccine confidence. How was vaccine confidence conceptualized for the purpose of this study? Please, provide a clear definition for vaccine confidence and some information about how both constructs are related.

Very important point – thank you. Originally, vaccine hesitancy was defined, by not confidence. This has now been amended/included in the second paragraph of the Introduction, and supported by previous research. We have also included a sentence in the final paragraph of the Introduction to suggest that, although they are related, they are also distinct (hence why the need to assess for confidence and hesitancy individually in this paper).

• The author(s) discussed historical events in the Philippines that may impact vaccine confidence and uptake. Medical mistrust could be an appropriate proxy to assess this theorized phenomenon.

Thanks – good point. We have now mentioned medical mistrust explicitly whilst discussing the influence of the Dengvaxia vaccine controversy (in the Introduction). We have also mentioned medical mistrust whilst talking about Social Cognitive Theory (SCT), a theory that we deemed appropriate in explaining which factors interact to predict vaccine-related hesitancy and confidence. Medical mistrust seemed fitting to include as a relevant outcome in this model/section.

• The introduction would be stronger if the author(s) include a discussion on factors known to be associated with vaccine hesitancy and confidence based on extant literature. Including that background information would help the reader better understand the selection of independent variables, like religion for instance, in the analysis.

Great point – thank you. We have now included extra sentences explaining our reasoning for some of the IVs included in the model. These include religion as per your suggestion, as well as the influence of living in rural/urbanised areas (lines 98-103). Whilst we are unable to touch on all IVs included in our model, we hope that this paragraph is sufficient in explaining how various factors may weaken confidence in vaccines. The remaining IVs are discussed in more depth in the Discussion.

• Besides age, what eligibility criteria was used to define the target population? Was language proficiency a factor? Was the survey written in English? After reading the Methods section, it is still unclear whether only unvaccinated Filipino and Malaysian residents were included in the study given that the aim of the study is to assess vaccine hesitancy and confidence levels in “unvaccinated individuals”.

Brilliant – thank you. We have now included extra sentences in the Methods section clarifying this – that is, that residents were aged 18 years old or older and required an understanding of the English language. We have also removed any mention that participants were ‘unvaccinated’ throughout the manuscript since we did not measure this in the survey (also discussed in the Discussion). The focus was on vaccine hesitancy and confidence only. Issues pertaining to these points are discussed in the Limitations.

• The “Measures” section could use further development. Due to the lack of contextual information, some of the categorization/coding of variable seem arbitrary and unclear. Was age treated as a continuous variable? If yes, that should be clearly stated. If not, how was it categorized? Religion is classified as “Christian/Buddhism/Muslim/Hinduism”. Is that an exhaustive list of potential religious affiliations in Malaysia and Philippines? Was there an “Other” category on the survey? How did you account for participants who do not practice any form of organized religion? In addition, the income variable is categorized as “sufficient vs. insufficient”. Was this subjectively reported by the participant? Otherwise, what income guidelines were used to determine if the participant’s income was sufficient?

These variables (age, religion, income) have now been explained in fuller detail in the Methods section. Age was indeed categorised, and religion included ‘other’ or ‘none’ categories. Finally, income was a continuous variable (1-4 Likert scale). We discuss how these variables were coded in the ‘data analysis’ section (lines 211-219).

• Relatedly, to provide more context, the author(s) should elaborate on how the variables were coded. Another example- was “social media users” posed as a simple yes/no question on the survey or was the variable coded as yes/no based a survey item that assessed frequency of social media use. Essentially, the author(s) should be clearer about how each variable was coded. Though some of the requested information is present in Table 1, it should also be discussed in the Methods section.

Thanks for this comment. We have explained each of the variables in more detail/in the Measures section, which now includes all options that were available to participants upon completion of the survey (in brackets). This section now contains four subsections: ‘Demographics,’ ‘Subjective social status,’ ‘Vaccine confidence and hesitancy,’ and ‘Digital health literacy.’ The “social media users” question has been explained in the ‘Demographics’ subsection, and includes how participants responded to this statement (i.e., no vs. yes).

• Author(s) should please provide a brief explanation of the MacArthur Scale of Subjective Social Status within the text of the manuscript.

Good point. We have now created a paragraph under the ‘Measures’ section dedicated to this scale (lines 152-160). We deemed it important to provide the reader with fuller context as to how subjective social status was measured, especially given the complexities of this scale from the perspective of the participant.

• Vaccine confidence and hesitancy were assessed with two questions. What is the source of the questions/items? Are the items validated? Are they from existing literature? Is there evidence to ascertain that the two questions/items posed on the survey are valid and reliable assessments of vaccine hesitancy and confidence? Also, how were the responses to these items coded for the analysis?

Great – thank you. These items were indeed adapted from the World Health Organization (WHO) survey. We talk more about how these responses were coded for analysis in the ‘Data Analysis’ section (lines 211-219), although we also address the important issues of using single items for measurement in the discussion/limitations section.

• Is there a scoring system for the DHLI? How are results from the DHLI interpreted?

Thanks for this point. There is no scoring system for the DHLI. However, each of the four 3-item subscales included are measured on a continuous Likert scale (1-4), which we discuss in more detail in the ‘Digital Health Literacy’ paragraph of the Measures. In fact, your point also reminded us to include the means, standard deviations, and reliability alphas for each of the subscales included. Thank you.

• Discussion- Lines 227-228 discusses the economic impact of the pandemic. This statement seems out of place given the focus of the paper.

Thanks. We have now removed this statement/sentence from the paragraph.

• Other limitations to this study not discussed in the paper. 1) This was an online survey meaning it is selective for Filipinos and Malaysians with access to internet who may be distinctly different from those who do not have access to the internet. 2) I have concerns about the validity and reliability of the items used to assess vaccine hesitancy and confidence. 3) Not all pertinent independent variables are accounted for (past experiences with vaccines, perception of risk/vulnerability, medical mistrust, vaccine knowledge).

Excellent – thank you. We realised as a result of this comment that our previous limitations section was unreasonably short. We have also discussed additional limitations to this study, including your points regarding representativeness of Filipinos and Malaysians without internet access, the validity of the items assessing vaccine hesitancy and confidence (indicating the need for further items to increase validity), and how additional variables were not accounted for due to space limitations. Thank you, again, for these suggestions.

**Reviewer #3:**

A) TITLE: This title requires minor modification and reviewer suggests "Sociodemographic Factors and Information-Seeking as predictors of COVID-19 Vaccine Hesitancy and Confidence in the Philippines and Malaysia" The inclusion of the words "factors" and "predictors" has defined the study design and statistical analysis involved and need not appear in the title. Again, observing that your abstract already has the word "predictors" establishes the coherence of the title and the abstract.

Thank you. This was in fact the manuscript’s previous title – I have a feeling that I may have neglected to update one of the submission forms with the new title. However, the title has since been amended to: **“COVID-19 Vaccine Hesitancy and Confidence in the Philippines and Malaysia: A Cross-sectional Study of Sociodemographic Factors and Digital Health Literacy.”** I hope that this is sufficient in summarising the study design and key outcomes.

B) ABSTRACT: The abstract has not stated the objective of the study in any form. This can be stated as; "This study sought to investigate the extent to which Sociodemographic Factors and Information-Seeking may predict the level of Confidence in COVID-19 Vaccines and the corresponding Hesitancy to accept vaccination among adult participants selected from population in the Philippines and Malaysia"

Many thanks for this. We have now amended the abstract and included our key aims as per your suggestion. This now leads nicely into the methodology. However, we reworded the aim to focus on “behavioural or environmental predictors” associated with COVID-19 hesitancy and confidence in order to represent the majority of predictors included in the model. Thank you again.

No mention of the method of instrument validity and reliability including constructs measured and how.

Thanks for pointing this out. We have now included the reliability alphas in all continuous variables included in the stud (in the Methods section). We have also cited/referenced any scales that have been used and/or validated in previous research. These include the DHLI, the MacArthur Scale of Subjective Social Status, and the individual items that we used to assess vaccine confidence and hesitancy.

The results reported in the abstract should be carefully articulated to provide clarity of groups being compared among other issues. For example, in line 45, the sentence "Results showed that Filipino (vs. Malaysian) participants indicated higher prevalence of COVID-19 vaccine hesitancy (56.6 vs. 22.9%, p =0.001). This statement does not convey sufficient clarity. If the two populations are being compared, then, the statement should be written in the conventional style; "Results showed that Filipino (56.6%) participants exhibited higher prevalence of COVID-19 vaccine hesitancy than Malaysians (22.9%) in the study (p <0.001)". Note, the p-value should be p<0.001, this is more conventional than p=0.001. It is observed that levels of confidence in the vaccine and information-seeking was not measured or reported in the abstract. This is an important omission.

Thank you. This sentence has now been included to improve clarity, and the p-values have now been tweaked. We have also touched on vaccine confidence and hesitancy in the Abstract (i.e., within the aims) so that the reader knows that they were measured. We have also included a brief reference to digital health literacy in the abstract (rather than information-seeking specifically, since I-S is a subscale of digital health literacy). This now reflects and accurately syncs with the manuscript’s title.

Although multivariate analysis was reported with Odds Ratio in the main study, outcomes of such analysis did not feature in the abstract. It would be interesting and revealing to report the ORs (Odds Ratio) for predictors between the two populations from line 52-56.

Great – the key findings and their associated Odds Ratios are now reported in the abstract. We tried to be as generous as possible with the findings included, especially within the realms of the word count.

C) INTRODUCTION: Editorial corrections in Line 69 is needed; "...the world’s population are yet to receive a single dose…". Correction is required because still and yet express the same meaning.

Good spot – thank you. This has now been clarified: “… 32.1% of the world’s population have not received a single dose of the COVID-19 vaccine as of July 2022.”

Note that the thesis in this study is centered on the statement in lines 84-86 "Thus, developing a deeper understanding of the factors associated with vaccine hesitancy and confidence will be crucial toward informing locally-tailored health promotion strategies.". Therefore, in this study at this time, it is not just understanding that is so much important but the explanations the study will provide based on appropriate scientific principles derived from epidemiology, behaviour theories responsible for the way the population respond to health threats and public health prevention and control.

This is true – thank you for pointing this out. We have now included a sentence stating how important it is to understand how specific populations respond to health threats and public health controls in the introduction. We have also included a brief paragraph in the implications section stating how important it is to understand how specific populations may be influenced by digital platforms, such as social media, at the start of the implications section. Thus, by including these sentences, we have emphasised on how important it is to “explain” who and how specific groups could respond to public health threats and misinformation. This then leads neatly into the implications.

The study has three important predictors, "sociodemographic factors", "information-seeking behaviour" and "confidence-also referred to as self-efficacy expectation with its associated outcome expectancy". These are constructs grounded in behaviour theories in the domain of Bandura's social cognitive theory, Information-motivation-behavioural skills model, and health literacy definitive of information typologies, that requires clarifications regarding how these constructs are able to provide the elucidation the study seeks. There is no theoretical foundation to sustain any argument the study may put up in the absence of these theoretical considerations. The absence of a theoretical framework has undermined the validity of the constructs to be measured in the study and any argument being put up in the global conversation related to COVID-19 vaccines and halting the spread of the virus. For instance, information-seeking has no theoretical characteristics of information and conceptual link to the definitions of information, nor health literacy reviewed. Therefore, it is very pertinent to have a paragraph somewhere in line 96 before statement of objective to place the theoretical and conceptual clarifications of likely underpinning dynamics in the web of interactions between the various constructs leading to the development of a conceptual framework.

Great! We have now included a paragraph discussing Bandura’s social cognitive theory (SCT) in the paragraph before the statement of objective(s) in the Introduction. Here, we have attempted to utilise the SCT to briefly illustrate (using examples) how and why specific factors may interact to produce hesitancy/confidence. In the same paragraph, we have also touched on why digital health literacy and social media are potentially important predictors of hesitancy/confidence, which leads up to the introduction of the SCT. We feel that it is already stronger for having included this – so thank you very much for this recommendation.

Note that the aim of the study is not clearly well stated, as it is absent in the abstract. Suggested revision: “The main aims of this research were to determine levels of hesitancy and confidence in COVID-19 vaccines among unvaccinated individuals in population of the Philippines and Malaysia, and to identify predictors significantly associated with these outcomes.”. It is worthy of note that the use of the word “influencing” cannot be ascertained in a cross-sectional study design despite the use robust statistical analysis.

Lovely – thanks. We have now included this sentence in the abstract and the introduction section right before the methods. We have also removed any mention of the word ‘influencing’ that may have been mentioned in the manuscript due to the study’s cross-sectional nature.

D) Methodological approach appears adequately conceived and implemented.

Thank you.

E) Similarly, the results appear adequately presented.

Thank you.

F) The discussion would have benefitted from reiterating the main aim of the study before presenting the implications of the data and concluding.

Great – thanks for this. We have included a sentence reiterating the study’s main aims at the end of the first paragraph in the Discussion. The paragraph now leads nicely into the interpretation of the study’s core findings in the paragraphs that follow.

G) The reference listings: Currency of literature cited is impressive but few online resources do not have dates accessed.

Thanks. Dates have now been added to the relevant references. Although, the majority of these references required digital object identifiers, so these have now been added. We have also tidied up the references section as a whole.
